# A New Superhard Phase and Physical Properties of ZrB_3_ from First-Principles Calculations

**DOI:** 10.3390/ma9080703

**Published:** 2016-08-22

**Authors:** Gangtai Zhang, Tingting Bai, Yaru Zhao, Yanfei Hu

**Affiliations:** 1 College of Physics and Optoelectronics Technology, Baoji University of Arts and Sciences, Baoji 721016, China; gtzhang79@163.com (G.Z.); scu_zyr@163.com (Y.Z.); 2 College of Mathematics and Information Science, Baoji University of Arts and Sciences, Baoji 721013, China; btt1120@163.com; 3 School of Science, Sichuan University of Science and Engineering, Zigong 643000, China

**Keywords:** ZrB_3_, structure prediction, superhard material, thermodynamic properties, 61.66.Fn, 61.50.Ah, 62.20.de

## Abstract

Using the first-principles particle swarm optimization algorithm for crystal structural prediction, we have predicted a novel monoclinic *C*2/*m* structure for ZrB_3_, which is more energetically favorable than the previously proposed FeB_3_-, TcP_3_-, MoB_3_-, WB_3_-, and OsB_3_-type structures in the considered pressure range. The new phase is mechanically and dynamically stable, as confirmed by the calculations of its elastic constants and phonon dispersion curve. The calculated large shear modulus (227 GPa) and high hardness (42.2 GPa) show that ZrB_3_ within the monoclinic phase is a potentially superhard material. The analyses of the electronic density of states and chemical bonding reveal that the strong B–B and B–Zr covalent bonds are attributed to its high hardness. By the quasi-harmonic Debye model, the heat capacity, thermal expansion coefficient and Grüneisen parameter of ZrB_3_ are also systemically investigated.

## 1. Introduction

Superhard materials are considerably used in various industrial applications due to their superior properties of low compressibility, thermal conductivity, refractive index, chemical inertness, and high hardness. Previously, it was generally accepted that the superhard materials are strong covalently bonded compounds formed by light atoms B, C, N, and O, such as diamond [1], *c*-BN [2], B_6_O [3], etc. These materials are expensive and rare, for they can be synthesized only under high-temperature and high-pressure conditions. Recently, it has been reported that the incorporation of light atoms (B, C, N and O) into heavy transition metals (TMs) with high valence electron densities provides a good candidate for designing new hard materials. The compounds formed by TM and light atoms usually possess high valence electron density and directional covalent bonds, and these covalent bonds are strong enough to inhibit creation and movement of dislocations, which greatly improve their mechanical properties and create high hardness. Based on this design criterion, the recent design of new intrinsically potential superhard materials has focused on TM borides, e.g., ReB_2_ [4], OsB_2_ [5], WB_4_ [6,7], CrB_4_ [8] and so on. The obtained results exhibit that these materials all have large bulk and shear moduli. Moreover, TM borides can be synthesized under ambient pressure, which results in a low-cost synthesis condition and is beneficial to their application. Thereby, these pioneering studies open up a new route for pursuing new superhard materials.

Up to now, in the Zr-B system, three binary phases [9,10], i.e., ZrB, ZrB_2_, and ZrB_12_, have been synthesized experimentally. Among them, ZrB with the NaCl-type cubic structure has a high melting point and thermal conductivity, and its thermal stability is in the temperature range from 1073 to 1523 K [9]. ZrB_2_ is an AlB_2_-type hexagonal structure and has properties of both ceramics and metals, and its melting temperature has been reported to be either 3313 [9] or 3523 K [11,12]. Meanwhile, ZrB_2_ also possesses high hardness, electrical conductivity, thermal conductivity, and chemical corrosion resistance [13,14]. ZrB_2_ ceramic products have been considerably used in making high-temperature structural and functional materials [15,16]. ZrB_12_ with an UB_12_-type cubic structure was formed by the peritectic reaction at *T* = 2303 K [11], and it presents a wide range of interesting characteristics such as high temperature resistance, superconductivity, and large hardness. The crystal structure and related physical properties of ZrB, ZrB_2_, and ZrB_12_ have been investigated theoretically [17,18,19,20,21,22,23,24,25,26]. By using the first-principles calypso algorithm for crystal structure prediction, Zhang et al. [27] predicted two orthorhombic *Cmcm* and *Amm*2 structures of ZrB_4_, and they also showed that these two phases are potentially superhard materials because of large shear modulus (229 and 236 GPa) and high hardness (42.8 and 42.6 GPa). Very recently, Chen et al. [28] systemically investigated the elasticity, hardness, and thermal properties of ZrB*_n_* (*n* = 1, 2, 12) by using the first-principles calculations of plane-wave ultra-soft pseudo-potential technology based on the density functional theory. However, there is no experimental report on the synthesis of zirconium triborides; besides, the theoretical research works of zirconium triborides are also few so far.

In this work, the crystal structures of ZrB_3_ are extensively investigated by the first-principles particle swarm optimization algorithm (PSO) on crystal structural prediction [29,30,31]. A stable monoclinic *C*2/*m* structure has been uncovered for ZrB_3_, which is much more energetically preferable than the earlier proposed FeB_3_-, TcP_3_-, MoB_3_-, WB_3_-, and OsB_3_-type structures in the pressure range of 0–100 GPa. The lattice parameters, total energy, formation enthalpy, phonon frequency, phase stability, elastic properties, density of states, and electronic localization function are then performed to study this novel monoclinic phase by using the first-principles calculations. To further study ZrB_3_, the thermodynamic properties are also investigated by the quasi-harmonic Debye model.

## 2. Computational Methods

To search for potential structure, the PSO technique in crystal structure analysis using the CALYPSO code [32] has been implemented at 0 GPa with one to four formula units (f.u.) in each simulation cell. The underlying ab initio calculations are performed using density functional theory within the Perdew–Burke–Ernzerhof (PBE) generalized gradient approximation (GGA) of the exchange-correlation energy, as implemented in the Vienna ab initio simulation package (VASP) [33]. The all-electron projector augmented wave (PAW) method [34] is employed with 2*s*^2^2*p*^1^ and 4*s*^2^4*p*^6^4*d*^2^5*s*^2^ treated as the valence electrons for B and Zr, respectively. Geometry optimization is performed using the conjugate gradient algorithm method with a plane-wave cutoff energy of 520 eV. The calculations are conducted with 11 × 7 × 4, 9 × 11 × 6, 3 × 11 × 10, 8 × 8 × 4, 8 × 8 × 5, and 13 × 13 × 7 for the predicted *C*2/*m*-ZrB_3_ structure and considered FeB_3_-type (No, 11, *P*2_1_/*m*), TcP_3_-type (No, 62, *Pnma*), MoB_3_-type (No, 166, *R*-3*m*), WB_3_-type (No, 194, *P*6_3_/*mmc*), and OsB_3_-type (No, 187, *P*-6*m*2) structures. For hexagonal structures, Γ-centered *k* mesh is used, and for other structures, appropriate Monkhorst-Pack *k* meshes [35] are used to ensure that all the total energy calculations are well converged to better than 1 meV/atom. The phonon calculation is carried out by using a supercell approach as implemented in the PHONOPY code [36,37]. Single-crystal elastic constants are calculated by a strain-energy approach, i.e., applying a small strain to the equilibrium lattice and fitting the dependence of the resulting change in energy on the strain. The bulk modulus, shear modulus, Young’s modulus, and Poisson’s ratio are derived from the Voigt–Reuss–Hill approximation [38]. The theoretical Vickers hardness is estimated by the empirical formula proposed by Chen et al. [39]. Moreover, the quasi-harmonic Debye model [40], which is constructed from the Helmholtz free energy at the temperature below the melting point in the quasi-harmonic approximation, is used to obtain thermodynamic properties of ZrB_3_.

## 3. Results and Discussion

**Figure 1:**
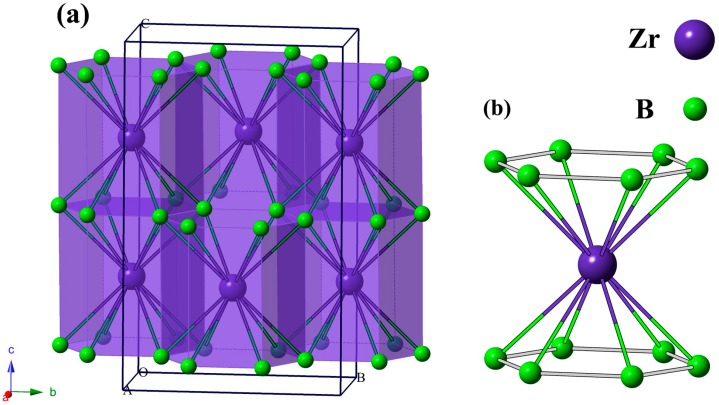
Crystal structure of the *C*2/*m*-ZrB_3_ phases. Large and small spheres represent Zr and B atoms, respectively. (**a**) Polyhedral view of the *C*2/*m* phase; (**b**) ZrB_12_.

Through the PSO technique, we perform a variable-cell structure prediction simulation for ZrB_3_ including 1–4 f.u. in the simulation cell at 0 GPa, and we successfully predict a novel monoclinic structure with space group *C*2/*m*, as depicted in Figure 1. For the monoclinic *C*2/*m* structure, it contains four ZrB_3_ in a unit cell with the equilibrium lattice parameters of *a* = 3.163 Å, *b* = 5.440 Å, *c* = 8.773 Å, α = γ = 90°, and β = 93.633°, in which three inequivalent Zr, B1, and B2 atoms occupy the Wyckoff 4*i* (0.4677, 0, 0.2994), 8*j* (0.8915, 0.1694, 0.0932), and 4*h* (0, 0.1668, 0.5) sites, respectively. Figure 1a presents the polyhedral view for the predicted *C*2/*m* structure, which shows an intriguing B–Zr–B sandwich stacking order along the crystallographic *c*-axis. In this structure, B atoms form parallel hexagonal planes, and each Zr atom is coordinated by 12 neighboring B atoms, forming edge-shared ZrB_12_ hexagonal columns which are connected by boron hexagonal planes. For each ZrB_12_, the calculated B–B distances are 1.809 (×4), 1.844 (×2), 1.822 (×4), and 1.816 (×2) Å, while the calculated Zr–B distances are 2.729 (×2), 2.563 (×2), 2.434 (×2), 2.472 (×2), 2.528 (×2), and 2.599 (×2) Å.

**Figure 2:**
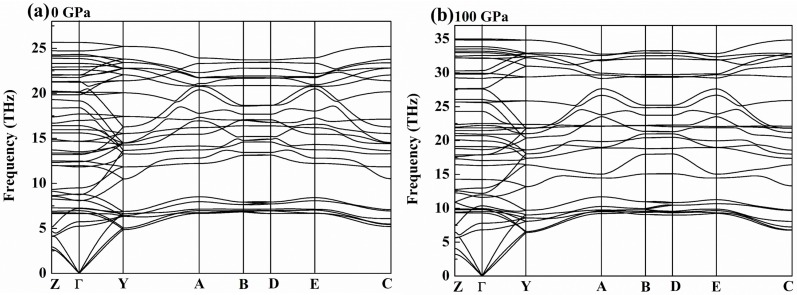
Phonon dispersion curves of the *C*2/*m*-ZrB_3_ at 0 GPa (**a**) and 100 GPa (**b**).

**Figure 3:**
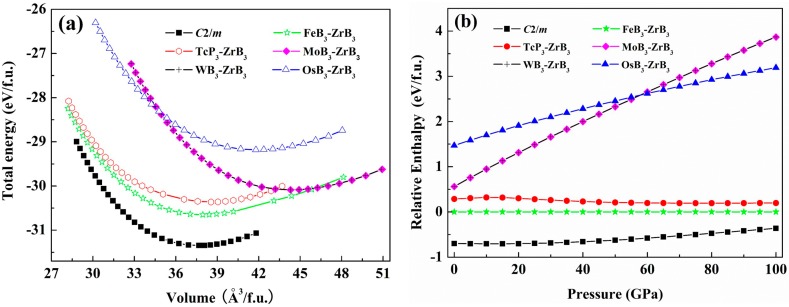
(**a**) Total energy versus f.u. volume for ZrB_3_ with six different structures; (**b**) Enthalpies of ZrB_3_ with six different structures relative to FeB_3_-ZrB_3_ phase as a function of pressure.

At zero temperature, a stable crystalline structure requires all phonon frequencies to be positive. Therefore, we calculate the phonon dispersion curves for the predicted *C*2/*m*-ZrB_3_ at 0 and 100 GPa, respectively. As shown in Figure 2, no imaginary phonon frequency appears in the whole Brillouin zone, indicating its dynamic stability at ambient pressure and high pressure. It is well known that the thermodynamic stability of a compound can be indicated by the energy of its most stable phase. Thus, we calculate the total energy per f.u. as a function of the volume for the predicted *C*2/*m* structure, as shown in Figure 3a. For comparison, the previously known five structures of FeB_3_, TcP_3_, MoB_3_, WB_3_, and OsB_3_ are also considered for ZrB_3_. As seen from Figure 3a, our predicted *C*2/*m* structure for ZrB_3_ has a lower energy minimum than the FeB_3_-, TcP_3_-, MoB_3_-, WB_3_-, and OsB_3_-type structures. This implies that the predicted *C*2/*m* structure is the ground-state phase at 0 GPa. Figure 3b shows the enthalpy curves of ZrB_3_ with six different structures relative to the FeB_3_-ZrB_3_ phase. One can see clearly that the predicted *C*2/*m* phase is much more energetically favorable than the previously proposed FeB_3_-, TcP_3_-, MoB_3_-, WB_3_-, and OsB_3_-type structures in the whole pressure range.

**Figure 4:**
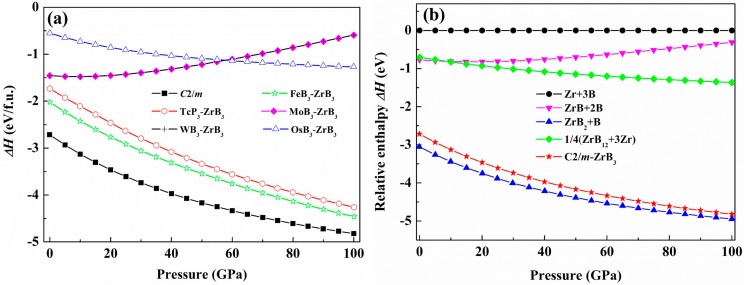
(**a**) The formation enthalpy-pressure curves for ZrB_3_ with six different structures; (**b**) The relative enthalpy-pressure curve of the *C*2/*m*-ZrB_3_ and its respective competing phases with respect to elemental Zr and B.

We also calculate the formation enthalpies of the considered structural candidates of ZrB_3_ in the pressure range of 0–100 GPa. The formation enthalpy of ZrB_3_ with respect to the separate phases can be examined by following the reaction route $\Delta H=H_{{\text{ZrB}}_{3}}-H_{\text{Zr}}-3H_{B}$, where ∆*H* represents the formation enthalpy and the hexagonal Zr and α-B are chosen as the reference phases. Figure 4a presents the calculated formation enthalpies of ZrB_3_ with different structures under pressure. It can be clearly seen from this figure that the predicted *C*2/*m* structure is the most stable phase of all the considered structures up to 100 GPa. All the considered structures are thermodynamically stable with a negative value of the formation enthalpy below 100 GPa. Among them, the predicted *C*2/*m* phase has the lowest formation enthalpy of −2.715 eV at 0 GPa. Therefore, the *C*2/*m* phase is more easily synthesized at ambient conditions, and meanwhile this also indicates that the predicted *C*2/*m* phase is more stable than the reference phase mentioned above. Figure 4b presents the relative enthalpy-pressure curves of *C*2/*m*-ZrB_3_ and its respective competing phases with respect to elemental Zr and B. Here we choose ZrB, ZrB_2_, and ZrB_12_ as the competing phases because they have been synthesized in experiments and are thermodynamically stable. From Figure 4b, the predicted *C*2/*m*-ZrB_3_ is much more energetically stable for decomposing into elements (Zr + B), compounds ZrB + B and ZrB_12_ + Zr in the pressure range from 0 to 100 GPa, whereas the competing phase ZrB_2_ + B is the most stable phase within the given pressure range. It is noteworthy that the relative enthalpy difference between the *C*2/*m*-ZrB_3_ and ZrB_2_ + B becomes smaller and smaller with the increasing pressure. This indicates that the ZrB_3_ may be the most stable phase against which to decompose into compounds ZrB_2_ + B when the pressure exceeds 100 GPa.

Mechanical stability is a necessary condition for the existence of a crystal, and the mechanical properties (elastic constants and elastic moduli) are important for potential technological and industrial applications. Accurate elastic constants help to understand the mechanical properties and also provide useful information for estimating the hardness of a material. By using a strain-energy method, we obtain the zero-pressure elastic constants (*C_ij_*) of the *C*2/*m*-ZrB_3_. The calculated elastic constants are listed in Table 1 along with the theoretical values and available experimental data of other zirconium borides, MoB_3_, WB_3_, RuB_3_, and OsB_3_. For a stable monoclinic crystal, the independent elastic stiffness tensor consists of 13 components, *C*_11_, *C*_22_, *C*_33_, *C*_44_, *C*_55_, *C*_66_, *C*_12_, *C*_13_, *C*_23_, *C*_15_, *C*_25_, *C*_35_, and *C*_46_, and its mechanical stability criteria can be given by:

(1) $$\begin{array}{c} C_{11}>0,C_{22}>0,C_{33}>0,C_{44}>0,C_{55}>0,C_{66}>0,\left[C_{11}+C_{22}+C_{33}+2\left(C_{12}+C_{13}+C_{23}\right)\right]>0,\\ (C_{33}C_{55}-C_{35}^{2})>0,(C_{44}C_{66}-C_{46}^{2})>0,(C_{22}+C_{33}-2C_{23})>0,\\ {}[C_{22}(C_{33}C_{55}-C_{35}^{2})+2C_{23}C_{25}C_{35}-C_{23}^{2}C_{55}-C_{25}^{2}C_{33}]>0,\\ g=C_{11}C_{22}C_{33}-C_{11}C_{23}^{2}-C_{22}C_{13}^{2}-C_{33}C_{12}^{2}+2C_{12}C_{13}C_{23},\\ \{2[C_{15}C_{25}\left(C_{33}C_{12}-C_{13}C_{23}\right)+C_{15}C_{35}\left(C_{22}C_{13}-C_{12}C_{23}\right)+C_{25}C_{35}\left(C_{11}C_{23}-C_{12}C_{13}\right)]-\\ {}[C_{15}^{2}(C_{22}C_{33}-C_{23}^{2})+C_{25}^{2}(C_{11}C_{33}-C_{13}^{2})+C_{35}^{2}(C_{11}C_{22}-C_{12}^{2})]+C_{55}g\}>0. \end{array}$$

**Figure 5:**
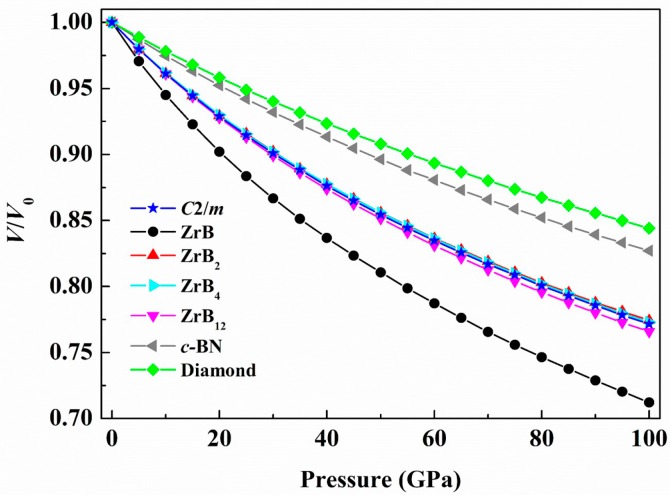
The calculated volume compression as a function of pressure for the *C*2/*m*-ZrB_3_ in contrast to ZrB, ZrB_2_, ZrB_4_, *c*-BN, and diamond.

As shown in Table 1, the elastic constants of the predicted *C*2/*m*-ZrB_3_ completely fulfill the elastic stability criteria for a monoclinic crystal, indicating that it is mechanically stable at ambient conditions. The values of *C*_11_, *C*_22_, and *C*_33_ for the new phase are all larger than 400 GPa, suggesting its strong incompressibility along the *a*, *b*, and *c* axis, respectively. Additionally, the values of *C*_11_ and *C*_22_ of the predicted *C*2/*m* phase are much higher than that of *C*_33_, which reflects that the bond strengths along the [100] and [010] directions are much stronger than that of the [001] direction. This is because the covalence of the B–B bond is higher than that of the Zr–B bond, as indicated by Figure 8. Thus, the relatively small *C*_33_ with regard to *C*_11_ and *C*_12_ is understandable. *C*_44_ is an important indicator for the hardness of a material. The large *C*_44_ value (207 GPa) of the predicted *C*2/*m*-ZrB_3_ indicates its relatively strong strength against the shear deformation. Using the obtained elastic constants, the polycrystalline bulk modulus *B* and shear modulus *G* are thus determined by the Voigt–Reuss–Hill approximation. The Young’s modulus *E* and Poisson’s ratio *v* are derived by the following formulas: *E* = 9*BG*/(3*B* + *G*) and *v* = (3*B* − 2*G*)/(6*B* + 2*G*). The calculated bulk modulus, shear modulus, Young’s modulus, and Poisson’s ratio of the *C*2/*m* phase together with the reference materials mentioned above are listed in Table 1. As seen from Table 1, the predicted *C*2/*m* phase has a larger bulk modulus of 238 GPa, which is larger than those of ZrB [17,18,19,28,41] and ZrB_12_ [26,28,43] but comparable to those of ZrB_2_ [17,20,21,28,42], ZrB_4_ [27], and other TMB_3_ (TM = Mo, W, Ru, Os) [45,46,47,48]. This indicates that the *C*2/*m* phase is difficult to compress. Moreover, the bulk modulus (*B* = 238 GPa) for *C*2/*m*-ZrB_3_ agrees well with that directly obtained from the fitting results (*B*_0_ = 237 GPa) of the third-order Birch-Murnaghan equation of states, which further verifies the reliability of our elastic calculations. To compare the incompressibility of the *C*2/*m*-ZrB_3_, other zirconium borides, *c*-BN, and diamond under pressure, the volume compressions as a function of pressure are shown in Figure 5. Clearly, all the considered zirconium borides except for ZrB have almost the same incompressibility due to their very close bulk moduli, but their incompressibility is lower than those of *c*-BN and diamond. The shear modulus of a material is a measure of the ability to resist shape change at a constant volume and it plays an important role in hardness compared with the bulk modulus. Interestingly, the shear modulus of the predicted *C*2/*m*-ZrB_3_ is almost the same as those of ZrB_2_ [17,20,21,28,42] and ZrB_4_ [27], suggesting that it is a potential superhard material. Except the bulk modulus and shear modulus, the Young’s modulus can also provide a good measure of the stiffness of materials. Generally speaking, the larger the Young’s modulus a material has, the harder it is to deform. Since the Young’s modulus of the *C*2/*m*-ZrB_3_ is similar to those of the ZrB_2_ [17,20,21,28,42] and ZrB_4_ [27], it is conceivable that the *C*2/*m*-ZrB_3_ is a superhard material. Further, Poisson’s ratio *v* is a crucial parameter to describe the degree of directionality for the covalent bonding. From Table 1, the *v* value of the *C*2/*m*-ZrB_3_ (0.14) is a little larger than that of ZrB_2_ [42], the same as ZrB_4_ [27], and much smaller than those of the other TM borides mentioned above [45,46,47,48], which means there is a strong degree of covalent bonding due to the presence of the planar six-membered ring boron network. To describe the brittleness or ductility of materials, Pugh [49] proposed an experimental criterion by the ratio *G*/*B*. The critical value which separates ductile and brittle materials is about 0.57. If *G*/*B* < 0.57, the material behaves in a ductile manner; otherwise, the material behaves in a brittle way. It can be seen from Table 1 that the *G*/*B* value for the predicted phase is 0.95, implying it is rather brittle nature. All the results strongly support that the *C*2/*m*-ZrB_3_ is a potential candidate for an ultra-incompressible and superhard material.

**Figure 6:**
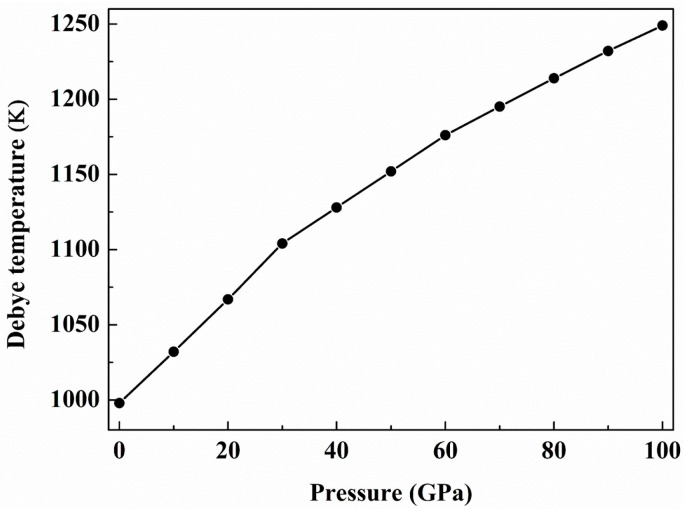
Debye temperature of the *C*2/*m*-ZrB_3_ as a function of pressure.

The Debye temperature is a very important parameter of materials, and it is closely related to many physical properties such as specific heat, elastic constants, melting temperature, hardness and so on [50]. It is used to differentiate between high- and low-temperature regions for a solid. If the temperature *T* > Θ_D_, all modes are expected to have the energy of *k*_B_*T*; if *T* < Θ_D_, the high-frequency modes are expected to be frozen, namely the vibrational excitations originate only from the acoustic vibrations. The Debye temperature Θ_D_ for our studied *C*2/*m*-ZrB_3_ is estimated from the average sound velocity (*v_m_*) by the following expression:

(2) $$\Theta_{D}=\frac{h}{k}{\left[\frac{3n}{4\pi }\left(\frac{\rho N_{A}}{M}\right)\right]}^{\frac{1}{3}}v_{m}$$

where *h* is Planck’s constant, *k* is Boltzmann’s constant, *N*_A_ is Avogadro’s number, *n* is the number of atoms per formula unit, *M* is the molecular mass per f.u., and ρ is the density. The average sound velocity *v_m_* is given by:

(3) $$v_{m}={\left[\frac{1}{3}\left(\frac{2}{v_{t}^{3}}+\frac{1}{v_{l}^{3}}\right)\right]}^{-\frac{1}{3}}$$

where *v_t_* and *v_l_* are the transverse and longitudinal elastic wave velocities of the polycrystalline material, which is determined by Anderson’s method [51]. Figure 6 presents the dependence of the Debye temperatures of the *C*2/*m*-ZrB_3_ on the pressure. At zero pressure and zero temperature, our calculated Debye temperature is 998 K for the *C*2/*m*-ZrB_3_, which is much larger than the values of the known ultra-incompressible RuB_2_ (780 K) [52], ReN_2_ (735 K) [53], ReO_2_ (850 K) [54], and ReB_2_ (858.3 K) [55]. Generally, the higher the Debye temperature materials possess, the larger their microhardness. Clearly, the Debye temperature becomes greater with increasing the pressure. This indicates that the pressure is in favor of the improvement of the hardness for the *C*2/*m*-ZrB_3_.

Due to the high bulk and shear moduli as well as the large Debye temperature for the predicted *C*2/*m*-ZrB_3_, the hardness calculation is of great importance. The hardness of a material is the intrinsic resistance to deformation when a force is loaded, which depends upon the loading force and the sample quality. The Vickers hardness of a material is estimated by [39]:

(4) $$H_{v}=2{\left(K^{2}G\right)}^{0.585}-3$$

where *G* is the shear modulus and *K* = *G*/*B* is the Pugh modulus ratio. Using this model, the estimated Vickers hardness of the *C*2/*m*-ZrB_3_ is 42.2 GPa, which exceeds the lower limit of superhard materials (40 GPa) and is comparable to the known superhard materials B_6_O (45 GPa) [3] and *c*-BN (48 GPa) [56]. Therefore, the *C*2/*m*-ZrB_3_ is considered to be a superhard material.

**Figure 7:**
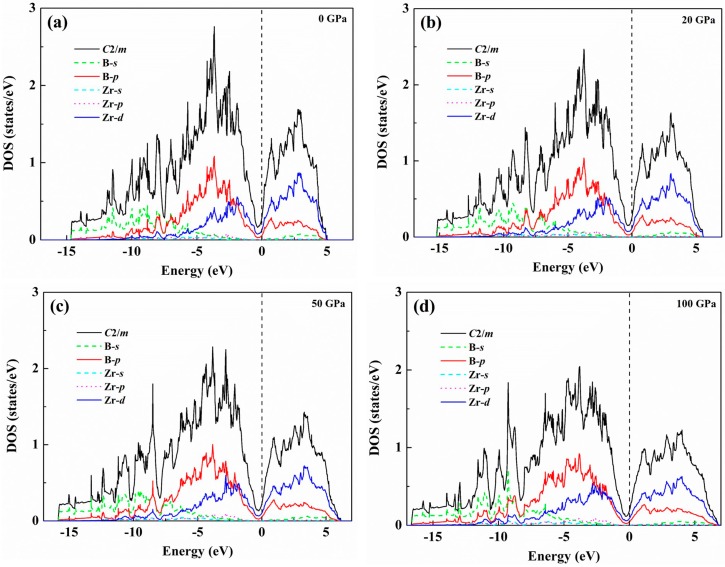
Total and partial density of states of the *C*2/*m*-ZrB_3_: (**a**) 0 GPa; (**b**) 20 GPa; (**c**) 50 GPa; and (**d**) 100 GPa. The vertical dashed line denotes the Fermi level *E*_F_.

The electronic structure and chemical bonding are key factors to further understand the origin of the mechanical properties of the *C*2/*m*-ZrB_3_. For this purpose, the density of states (DOS) and bond characteristics are calculated, and the corresponding results are analyzed here. Figure 7a presents the total and partial density of states of the *C*2/*m*-ZrB_3_ at 0 GPa, where the vertical dashed line denotes the Fermi level (*E*_F_). As seen from this figure, the *C*2/*m*-ZrB_3_ shows a metallic behavior because of the finite electronic DOS at the Fermi level. From the partial DOS, it exhibits that the peaks below −11 eV are mainly attributed to B-*s* and B-*p* states with slight contributions from Zr-*s*, Zr-*p*, and Zr-*d* states. The states above −11 eV largely come from Zr-*d* and B-*p* orbitals with small contributions of B-*s*, Zr-*s*, and Zr-*p*. Moreover, the partial DOS profiles of Zr-*d* and B-*p* have a very similar shape in the range from −11 to 0 eV, which indicates the significant hybridization between these two orbitals. This hybridization also reflects a strong covalent interaction between the Zr and B atoms. On the other hand, the DOS profile near *E*_F_ mainly originates from the 4*d* state of Zr. Another typical feature of DOS is that there is a deep valley, namely the pseudogap around *E*_F_, which is regarded as the borderline between the bonding and antibonding states [57,58,59]. Significantly, the pseudogap appears below the *E*_F_ in the *C*2/*m*-ZrB_3_, indicating the *s*-*p* and *p*-*d* bonding states started to be saturated. The nearly full occupation of the bonding states, and without filling the antibonding states, results in the high bulk modulus, large shear modulus, and small Poisson’s ratio, and also increases the structural stability of the *C*2/*m*-ZrB_3_. To further understand the changes of the total and partial DOS under pressure, Figure 7b–d present the total and partial DOS of the *C*2/*m*-ZrB_3_ at 20, 50, and 100 GPa, respectively. Compared with the case for 0 GPa (see Figure 7a), the shapes of the total and partial DOS are slightly changed with the increase of the pressure. This indicates that the *C*2/*m*-ZrB_3_ structure is still stable under pressure up to 100 GPa. Traditionally, the stability of a solid is closely associated with the DOS at the Fermi energy. According to our calculations, the DOS values at the Fermi level are 0.294 eV for 0 GPa, 0.263 eV for 20 GPa, 0.227 eV for 50 GPa, and 0.186 eV for 100 GPa. Therefore, the DOS value at the Fermi level decreases with increasing the pressure. Based on the previous reference report [60], there is a principle stating that the DOS at the Fermi level would be hopefully smaller in an energetically-favored structure. This means that the applied pressure is in favor of the stability of ZrB_3_. In addition, one can see from Figure 7 that the total DOS reveals a slight broadening with the enhancement of the pressure. All these results may be explained by the variations of the spacing between atoms.

**Figure 8:**
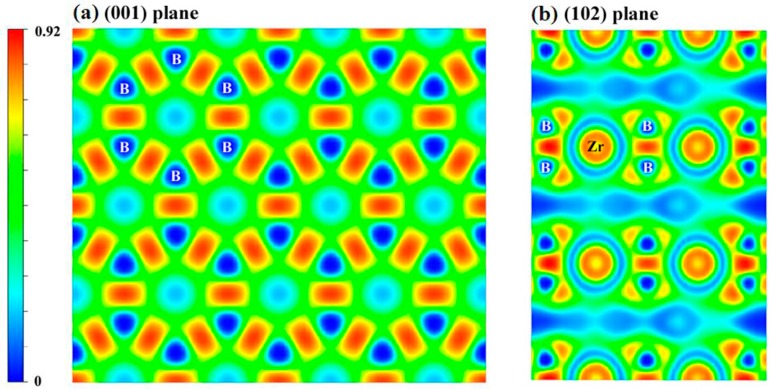
Contours of ELF of the *C*2/*m*-ZrB_3_ on the (001) (**a**) and (102) (**b**) planes at 0 GPa.

To gain deeper insight into the bonding character of the predicted *C*2/*m*-ZrB_3_, we calculate the electronic localization function (ELF), which is based upon a topological analysis related to the Pauli exclusion principle. The ELF is a contour plot in real space, in which different contours have values from 0 to 1. The upper limit ELF = 1 shows the perfect localization property of covalent bonds or lone pairs (filled core levels), whereas the lower limit ELF = 0 is typical for a vacuum (no electron density) or areas between atomic orbitals. ELF = 0.5 corresponds to the perfect free-electron gas, with values of this order meaning regions with bonding of a metallic character. Note that ELF is not a measure of electron density but a measure of the Pauli principle, and it is used to distinguish metallic, covalent, and ionic bonding. Figure 8 presents the calculated ELF contours of the predicted *C*2/*m*-ZrB_3_ phase on the (001) and (102) planes. Because of the high ELF value between the two adjacent B and B atoms, as shown in Figure 8a, there is the existence of a strong covalent B–B bonding within the planar six-membered ring unit. Meanwhile, one can see from Figure 8b that the large ELF value between the Zr and B atoms implies the partially B–Zr covalent bonding interaction in the *C*2/*m*-ZrB_3_ phase. Therefore, the strong covalent interaction between B–B bonds and B–Zr bonds is the major reason for its high hardness and stability.

**Figure 9:**
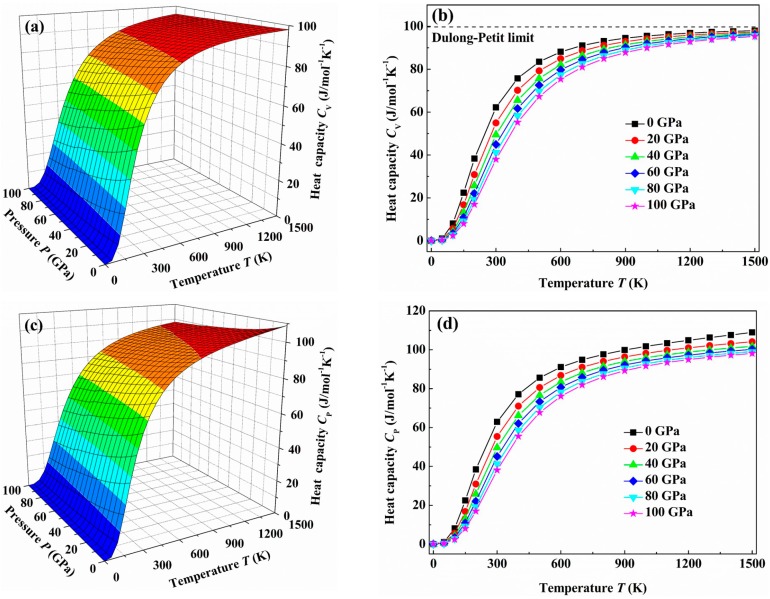
The temperature dependences of the heat capacity at constant volume *C*_V_ and heat capacity at constant pressure *C*_P_ at different pressures for the *C*2/*m*-ZrB_3_: (**a**) *C*_V_ contours; (**b**) *C*_V_-*T*; (**c**) *C*_P_ contours and (**d**) *C*_P_-*T*, respectively.

The investigation on the thermodynamic properties of solids at high temperature and high pressure is an interesting topic in materials science. Therefore, we investigate the thermodynamic properties of the *C*2/*m*-ZrB_3_ in the temperature range from 0 to 1500 K, where the quasi-harmonic model remains fully valid and the pressure effect is also investigated in the pressure range from 0 to 100 GPa. By applying this model, we can get the variations of the lattice heat capacity *C*_V_ or *C*_P_, the thermal expansion coefficient α, and Grüneisen parameter γ with temperature and pressure. Figure 9 presents the temperature dependences of the heat capacity at constant volume *C*_V_ and the heat capacity at constant pressure *C*_P_ at different pressures, respectively. As seen from Figure 9, when the temperature is lower than 400 K, the difference of between *C*_V_ and *C*_P_ is very small. This can be explained by the relation between *C*_P_ and *C*_V_, i.e., *C*_P_ *=* *C*_V_ *+* *TVB*α^2^ [61], since the difference between the *C*_P_ and *C*_V_ is mainly determined by α^2^ for the case of low temperature. Therefore, when the thermal expansion coefficient α becomes small, e.g., the temperature *T* decreases and the pressure *P* increases, the *C*_P_-*C*_V_ difference also becomes small. On the other hand, the *C*_V_ and *C*_P_ increase rapidly with the temperature at low temperature. This rapid increase is simply due to the exponentially increased number of excited phonon modes. However, when the applied temperature is considerably high, due to the anharmonic effect, the *C*_P_ is different from the *C*_V_. The former is proportional to *T* at high temperature, while the latter reaches a constant value which is the so-called Dulong-Petit limit (*C*_V_(*T*) ~ 3*R* for monoatomic solids). Moreover, as the pressure keeps constant, the *C*_V_ and *C*_P_ increase with the temperature. As the temperature keeps constant, *C*_V_ and *C*_P_ decrease with the pressure. This shows that the temperature has a more significant influence on the heat capacity than the pressure.

**Figure 10:**
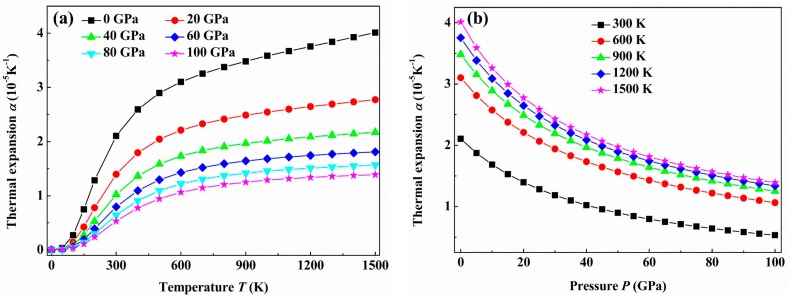
Temperature (**a**) and pressure (**b**) dependence of the thermal expansion coefficient α for the *C*2/*m*-ZrB_3_.

Figure 10 plots the changes of the thermal expansion coefficient α for the *C*2/*m*-ZrB_3_ with temperature and pressure. As shown in Figure 10a, the thermal expansion coefficient α increases sharply at low temperatures, especially for the case of 0 GPa, and then gradually approaches a linear increase at high temperature, finally becomes moderate at much higher temperature. In terms of α ~ *C*_V_/*B* [61], the fast increase of α at low temperature is mainly due to that of *C*_V_, and the linear increase of α at high temperature is attributed to that of bulk modulus *B* (*C*_V_ is saturated to the Dulong-Petit limit at high temperature). In addition, the effect of the pressure on α is very small for the case of low temperature, whereas the effect is clearly enhanced at sufficiently high temperature. When the pressure increases, α reduces drastically for a given temperature and the effect of the temperature on it becomes unnoticeable. From Figure 10b, one can see clearly that α is almost close to a constant value at high temperature and pressure. All these results are in good agreement with those of many kinds of materials by applying the Debye theory, such as OsB_4_, FeB_4_, and TcN [62,63,64].

**Figure 11:**
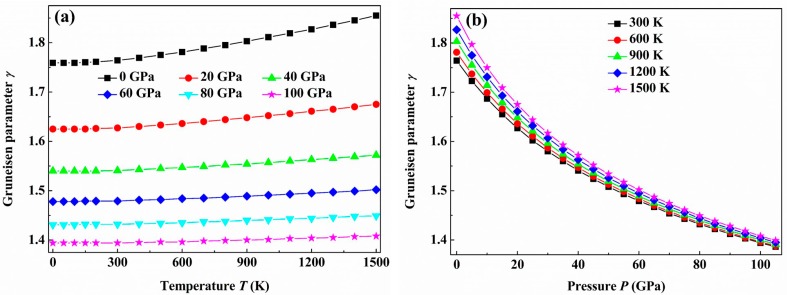
Temperature (**a**) and pressure (**b**) dependence of the Grüneisen parameter γ for the *C*2/*m*-ZrB_3_.

The Grüneisen parameter γ can describe the anharmonic effects in the vibration of a crystal lattice, and it is commonly used to characterize and explore the thermodynamic behavior of a material at high temperature and high pressure, such as the thermal expansion coefficient and the temperature dependence of phonon frequencies and line-widths. For this purpose, we investigate the dependences of the Grüneisen parameter γ for the *C*2/*m*-ZrB_3_ with the temperature and pressure, and the corresponding results are given in Figure 11. As seen from Figure 11a, for a given pressure, γ almost remains the same at the low temperature of ≤300 K. As the applied temperature is higher than 300 K, γ tonelessly increases with temperature for the given pressure. In addition, one can find from Figure 11b that γ decreases nearly exponentially with the pressure for a given temperature. As the applied pressure increases, the temperature has a much weaker influence on γ than the pressure.

## 4. Conclusions

In summary, a novel monoclinic *C*2/*m* structure is unraveled to be the ground-state structure for ZrB_3_ via the PSO algorithm combined with first-principles calculations. The phonon dispersion and elastic constant calculations have verified that the *C*2/*m*-ZrB_3_ is dynamically and mechanically stable. The formation enthalpy-pressure relationship shows that this new phase is energetically superior to the previously proposed FeB_3_-, TcP_3_-, MoB_3_-, WB_3_-, and OsB_3_-type structures within the pressure range of 0–100 GPa. In addition, the high bulk modulus, large shear modulus, and small Poisson’s ratio of the predicted *C*2/*m* phase indicate that it is a promising low-compressibility material. Based on the calculated Vickers hardness (42.2 GPa) for the *C*2/*m* phase, it is a potential superhard material. The more detailed analyses of the electronic structure and electronic localization function have further demonstrated that the strong covalent B–B bonding and B–Zr bonding are the main driving force for the incompressibility and hardness for *C*2/*m*-ZrB_3_. By using the quasi-harmonic Debye model, some fundamental thermodynamic properties, such as the heat capacity, thermal expansion coefficient, and Grüneisen parameter are estimated in the pressure range of 0–100 GPa and the temperature range of 0–1500 K. We hope that the present theoretical work will stimulate further experimental research on this material in the future. 

## Figures and Tables

**Table 1:** The calculated elastic constants *C_ij_* (GPa), bulk modulus *B* (GPa), EOS fitted bulk modulus *B*_0_ (GPa), shear modulus *G* (GPa), Young’s modulus *E* (GPa), *G*/*B* ratio, Poisson’s ratio *v*, Debye temperature Θ_D_ (K), and hardness *H_v_* (GPa) for the *C*2/*m*-ZrB_3_.

Structure	Work	*C*_11_	*C*_22_	*C*_33_	*C*_44_	*C*_55_	*C*_66_	*C*_12_	*C*_13_	*C*_23_	*B*	*B_0_*	*G*	*E*	*G*/*B*	*ν*	Θ_D_	*H_v_*
*C*2/*m*-ZrB_3_	this work	576	564	466	207	244	250	45	115	109	238	237	227	517	0.95	0.14	998	42.2
*Fm*-3*m*-ZrB	this work	363	-	-	60	-	-	60	-	-	161	160	88	223	0.55	0.27	529	10.5
	theory [17]	282	-	-	111	-	-	100	-	-	161	-	103	254	-	0.24	-	-
	theory [18]	350	-	-	43	-	-	70	-	-	163	-	71	186	-	0.31	-	5.5
	theory [19]	353	-	-	51	-	-	64	-	-	160	-	78	202	-	0.29	-	11.8
	theory [28]	274	-	-	42	-	-	51	-	-	125	-	63	162	0.50	0.28	450	7.9
	experiment [41]	-	-	-	-	-	-	-	-	-	148	-	-	244	-	0.23	-	-
*P*6/*mmm*-ZrB_2_	this work	565	-	432	252	-	255	54	121	-	239	238	233	527	0.98	0.13	930	44.1
	theory [17]	504	-	427	241	-	-	91	112	-	229	-	211	484	-	0.15	-	-
	theory [21]	551	-	436	252	-	-	65	121	-	239	-	229	520	-	0.14	921	-
	theory [28]	502	-	374	225	-	229	43	85	-	199	-	211	468	1.06	0.11	884	43.5
	experiment [20]	581	-	445	240	-	261	55	121	-	245	-	243	554	-	-	-	-
	experiment [42]	-	-	-	-	-	-	-	-	-	220	-	225	502	-	0.11	910	
*Amm*2-ZrB_4_	theory [27]	554	576	454	223	243	254	50	122	113	241	-	229	522	0.95	0.14 *^a^*	-	42.6
*Fm*-3*m*-ZrB_12_	this work	412	-	-	251	-	-	144	-	-	233	233	195	458	0.84	0.17	933	32.5
	theory [26]	413	-	-	244	-	-	141	-	-	232	-	193	453	0.83	0.174	1206	-
	theory [28]	420	-	-	249	-	-	123	-	-	222	-	202	466	0.91	0.15	1231	35.4
	experiment [43]	443	-	-	265	-	-	129	-	-	234	-	-	-	-	0.23	1260	-
	experiment [44]	-	-	-		-	-	-	-	-		-	-	-	-	-	1260	-
*Pnma*-RuB_3_	theory [45]	497	462	471	224	219	187	111	229	-	267		185	451	0.69	0.219	-	26.3
*R*-3*m*-MoB_3_	theory [46]	602	-	420	247	-	-	106	166	-	276	277	222	526	0.80	0.18	-	31.8
*P*6_3_/*mmc*-WB_3_	theory [47]	656	-	479	277	-	-	-	-	-	291	-	252	588	-	0.168	-	42
*P-*6*m*2-OsB_3_	theory [48]	525	-	751	186	-	-	125	221	-	317	-	195	485	0.62	0.245		36.9
*P*2_1_/*m*-OsB_3_	theory [45]	674	525	584	135	291	128	97	247	-	303	-	186	463	0.61	0.245		25.1

*^a^* Our calculated result from Reference [27] on the basis of *v* = (3*B* − 2*G*)/(6*B* + 2*G*).
